# Supplementary material for: Message framing and COVID-19 vaccine acceptance among millennials in South India
Source: PLoS One. 2022 Jul 8;17(7):e0269487. doi: 10.1371/journal.pone.0269487 (PMC9269925; doi:10.1371/journal.pone.0269487)
Supplement: Attachment — Submitted filename: Response to Reviewers.docx [file pone.0269487.s001.docx]

**Article Corrections after Review: PONE-D-22-05789**

**Note to Reviewers:** Dear Reviewers, we the authors of this study are indebted to you for giving us constructive comments for corrections. Upon going through all your comments and addressing them, we see this paper have significantly improved in quality. We thank you for all your comments and have addressed them point-by-point in this document for your review.

**Title: Message Framing and Covid-19 Vaccine Acceptance among Millennials in South India**

**Reviewer A Comments**:

This paper investigates the role of variables from the Theory of Planned Behavior and message frames (gain-framed vs. loss framed) in vaccination intentions among millennials in South India. I do believe the research is very interesting, relevant, and timely. I was very excited to read the paper based on the preview from the abstract. This research provides an important contribution to the growing literature on health communication during emerging infectious disease. However, despite the overall strength of the topic of this paper and the analyses conducted, the paper itself needs extensive revision across all sections. I’ve provided some commentary to hopefully help in this undertaking.

**Strengths of the Paper**

1. I appreciated the use of headers in the literature review- they were very helpful for following along

2. It was nice to see the authors report psychometric properties of constructs (assessing for different types of validity and factor loadings). I don’t think this practice is done enough, and it was a pleasant surprise and a particular strength of this manuscript.

3. Excellent job including rationale and description for decisions made about analyses.

| **#** | **Specific Comments** | **Corrections Made** |
| --- | --- | --- |
| 1 | Abstract  There are a couple of typos-  a. “intention to be vaccination” (line 6)  b. “in both message framing” (line 14)  c. “message framing to user behavioural intentions” (line 20) | Thank you. We have corrected the typos and checked through the abstract. |
| 2 | Authors reference “individual” attitudes on line 7, but then only refer to “attitude” on line 13. I’d keep this consistent, or explain what you mean by individual attitudes at first. I assumed that you investigated multiple different types of attitudes (e.g., cognitive vs. affective or about different health behaviors relevant to COVID-19), but when you refer to “attitude” generally later on when reporting results in the abstract, I wasn’t so sure. It might even be helpful to specify the type of attitude (e.g., attitude toward vaccination?). | Thank you for the suggestion, we agree with the idea that we specify that we are looking at attitudes towards COVID 19 vaccination. We have made this change. |
| 3 | Introduction  1. First paragraph: you cite various studies of research conducted in India- were all these studies correlational? I’m just assuming this because you used the term “associated with,” but I want to double check. This sounds like a major gap in the literature. If there is mixed methodology, it would only help to demonstrate that across a range of methodology, people who reported higher vaccine hesitancy also indicated concerns about vaccine safety, rumors, controversies about adverse effects; inadequate knowledge about vaccine benefits; and apprehension based on immunization cost and conflict with traditional cultural beliefs. Generally, I would suggest re-writing this sentence to make it clearer that these are reasons for why people demonstrated vaccine hesitancy about childhood immunization | Thank you for this observation. We have rectified this. The sentence now reads as follows:  “Various studies in India (S. & P., 2020; Sikder et al., 2021; Agrawal et al., 2020; Wagner et al., 2021) revealed that vaccine safety, rumours and controversies in terms of the adverse effects following immunisation; inadequate knowledge about vaccine benefits, costs and traditional cultural beliefs have been cited as major reasons for vaccine hesitancy in the domain of childhood immunisation.” |
|  | 2. Second paragraph: you reference “level of severity and the extent to which the pandemic is likely to affect the individual” – I’m a bit confused what exactly you are referencing, but it does sound like you are referencing risk perceptions, in which case I would suggest you state and describe this followed by a citation. | We have now included citations for this sentence:  “It can also be debated that the level of severity and the extent to which the pandemic is likely to affect the individual would serve as a good predictor to assess their intention to get vaccinated (Al-Mohaithef, 2020; Wang et al., 2021)” |
|  | 3. Second paragraph: “of which was the focus on vaccine hesitancy” is confusing… I’d suggest re-writing this sentence. Was it that one of the four critical areas that they suggested targeting was vaccine hesitancy, or they all were about targeting vaccine hesitancy? Also has there been research to test the efficacy of these four critical areas directly? It’s not clear if the subsequent sentences were about testing these critical areas directly? Also what do you mean by influencers? | Thank you for pointing this out. We have now rephrased the sentence as follows:  “The Ministry of Health and Family welfare in India identified four critical areas to be targeted as part of the communication strategy for COVID 19 vaccination- vaccine introduction, hesitancy, eagerness and following COVID appropriate behaviours. The aim was to identify messages and key messengers to disseminate information about the safety and efficacy of the vaccines to not just the traditionally resistant/hesitant groups but also to the general public at different levels to build trust (MoHFW, 2020). However, despite the holistic approach on communication strategies, COVID 19 vaccine hesitancy prevails in India.” |
|  | 4. Second paragraph: you end the last sentence “which could have been avoided.” This came across as somewhat abrupt, and I would recommend removing it and starting a new sentence to get across your point (i.e., it is necessary to test how health communications can be used as part of interventions to target vaccine hesitancy in the context of covid-19) | We agree, the sentence is rewritten as follows for better clarity.  In fact, a study that reported the findings of an online survey called the “Covid Symptom Survey” among Facebook users revealed that a significant portion of the participants (29%) were hesitant to get vaccinated and the reason was was mainly attributed to the safety of the vaccines (Chowdhury et al., 2021). This necessitates the need to understand how health communications can be effectively used as part of interventions to mitigate vaccine hesitancy. |
|  | 5. Third paragraph: it would be helpful if you gave examples immediately following your description of loss and gain framed messages. | Thank you. We have modified the paragraph accordingly. |
|  | 6. Third paragraph: could you include a transition before bringing up prospect theory? It is unclear to me how one is related to the other, particularly since prospect theory derives from the field of behavioral economics, and Gallagher and Updegraff are psychologists (and the work you cite more largely on persuasive messaging comes from the field of psychology). I see that you describe prospect theory and then bring in an implication based on gain/loss framing and prospect theory, but it doesn’t come across clearly. Examples might be helpful. | Thank you for the observation. We have cited Kahneman and Tversky’s paper to bring in the Behavioural Economics angle with more clarity. The sentence now reads as follows:  Along the same lines, the work on Prospect Theory reiterates that substantially identical information may have significantly different effects on people's decisions based on how it is framed (Kahneman & Tversky, 1979; Gallagher & Updegraff, 2011). For instance, two completely identical messages with respect to their subject matter differ only on how they have been framed (one that highlights the positive outcome while the other the negative outcome) is perceived differently. This implies that effective gain-framed messages emphasise the advantages of taking a risk, loss-framed messages, on the other hand, emphasise the costs of not taking a risk. |
|  | 7. Fourth paragraph: the previous studies you focus on in this paragraph seem out of place. I’d move them to your literature review, or to your second paragraph where you discuss other studies about covid in India. | Thanks for the suggestion. We have moved it to the second paragraph. |
| 4 | Literature Review  1. Do you have rationale for specifically targeting millennials for this research? | The main reason for targeting millennials is based on the findings of the following studies.  It is observed in previous studies that young adults are more likely to perceive vaccinations as harmful and unsafe than older adults (Anderson, 2015; Soares et al., 2021). Moreover, millennials largely turn to social media communication for pandemic related information (Beaudoin et al., 2021) which may shape negative views or misinformation towards vaccinations, emphasising the importance of effective persuasion messages. |
|  | 2. Theoretical Framework: you present the aim of your study within the literature review on your theoretical framework- it would be helpful to read your study aim earlier. | The aim of the study has been moved to the last paragraph of the introduction section. |
|  | 3. Perceived threat towards COVID-19- I don’t see a citation or explanation for why the rising cases could lead to increased perceived risk or threat. Indeed, participants could instead perceive lower threat or risk if they were engaging in defensiveness, which is a common response to threatening health information. | Thank you. We have added a citation to support the statement. Since we intend to measure the intention to take a vaccine, which is a form of defense against the virus, how people's risk perception has changed overtime is also studied.  The rapid increase in COVID 19 cases has been perceived as a health risk and threat (Kanovsky & Halamova, 2020). However, the prolonged nature of the pandemic and lower fatality rate makes people become optimistically biased of their chances of contracting the virus. This would not only lower their risk perception towards the virus, but also may make them hesitant to take up the vaccination (Chu, 2021). |
|  | 4. Social norms towards COVID-19 vaccine- you refer to the types of social norms as injunctive and descriptive in this paragraph, but then refer to them as direct/indirect social norms in your abstract. This is confusing. | We have modified the paragraph to better clarity this:  Social influence falls under two categories; direct and indirect. Direct social influence is a consequence of an individual swaying another individual’s opinion first hand, often through means of persuasion or manipulation. Indirect social influence exhibits subtle psychological proclivities in which an individual’s opinion or conduct is affected due to the information available on the other individual’s actions (Mavrodiev, Tessone and Schweitzer, 2013). ("Arguments of Direct and Indirect Social Influences", 2020). Therefore, injunctive norms are classified as indirect norms and descriptive norms as direct norms. |
|  | 5. Framing effects and TPB- I would avoid use of language like “it is clear” and rather offer assessment on whether there is strong evidence in support of the theory. In the same section you also refer to an “inability of past literature to produce conclusive results of framing” and so there is a contradiction in how you set this paragraph up. | Thank you for this suggestion. The idea that we were looking to convey was that while message framing is effective, literature seems inconclusive on whether loss or gain frames fare better. Apologies for the unclear language previously used; we have modified the sentences to give it more clarity.  “The inability of past literature to produce conclusive results on the debate between loss vs gain framed messages has made it necessary to probe into the role of these social cognitive constructs in relation to message framing and intention to behave in the context of the COVID-19 vaccine uptake.” |
| 5 | Hypotheses:  1. You reference a “positive impact” in your hypotheses, but it would be helpful to see written out exactly what that means  2. In H6, what is the significant difference that you hypothesized (if you did hypothesize specific differences a priori) | Thank you for the suggestion. We have now modified the hypotheses as follows:  Hypothesis 1 (H1). Attitudes play a significant role in predicting the intention to get vaccinated.  Hypothesis 2 (H2). Direct social norms play a significant role in predicting the intention to get vaccinated.  Hypothesis 3 (H3). Indirect social norms play a significant role in predicting the intention to get vaccinated.  Hypothesis 4 (H4). Perceived behavioural control plays a significant role in predicting the intention to get vaccinated.  Hypothesis 5 (H5). Perceived threat of contracting COVID 19 plays a significant role in predicting the intention to get vaccinated.  With respect to H6, we did not hypothesize a specific difference a priori, i.e only hypothesized a difference in messages framed differently (loss vs gain frame) based on the literature. We could not claim the advantage of one frame over the other since the literature provided mixed evidence. Hence we forwarded a neutral hypothesis to test for difference. |
|  | Materials and Methods  1. Did you pilot test your messages? If not, why not? Also, it’s unclear why you write India’s CDC in parentheses next to the messages. Is it because the information itself was from the CDC? In your text it sounds like the messages were created by the authors of the study. Clarification on whether this text was taken directly from the CDC (perhaps with only slight modification for the gain/loss framing?) or were created based on information from the CDC is helpful to know. | Yes, the messages were first tested among a small sample. A few changes were made to the messages based on the suggestions of the respondents for better clarity. The messages were framed based on the information available on the CDC website and Ministry of Health and Family Welfare. They were slightly tweaked to be presented as positive and negative messages. Apologizing for the confusion caused by putting the sources together, we have separated them. Link to the source website is given below for reference.  https://www.cdc.gov/coronavirus/2019-ncov/vaccines/vaccine-benefits.html#:~:text=Getting%20vaccinated%20against%20COVID%2D19,ages%205%20years%20and%20older. |
|  | 2. You reference “usable responses” for each condition in your study. What were the reasons for exclusion or eligibility? | Two manipulation check items were included in both questionnaires to ensure that the respondents read the frames. Inappropriate responses to the manipulation check questions were not included in the final data used for analysis. |
|  | 3. You mention sampling from individuals who are not vaccinated, but are the necessarily vaccine hesitant? Or perhaps they haven’t had an opportunity to be vaccinated yet? Data collection was carried out between August-September 2021- I’d recommend contextualizing when it was that the vaccine was made available and more information about access to the vaccine. I’m seeing later in the limitations section that WHO had not given approval for the vaccines, and so I think this point should be made earlier in regard to your sample… is it still appropriate to characterize the sample as vaccine hesitant if the vaccines were not even approved? | We have added the details of when the vaccines were made available to 18 years and above in India.  The Indian government approved vaccination for those aged 18 years and above on 1st May 2021. At the time of the study, the Indian made vaccine COVAXIN was pending to get approval by the WHO. The other vaccine available was the ChAdOx1-S, manufactured by the Serum Institute of India which was given approval for emergency use. |
| 6. | Results  1. I’m seeing many of the analyses/results are described in present tense, rather than past tense. | Apologies for this error, we have rectified this mistake. |
| 7. | Discussion  1. You bring up loss aversion almost immediately, but this phenomenon was not brought up earlier in the study- rather, you brought up prospect theory from the field of behavioral economics. How do these ideas relate?  2. You bring up other relevant research, but the discussion is very brief. I would like to see more of a description and integration of the studies you bring up (for example, an important point to consider- were these studies also in the context of COVID-19 vaccination, or vaccination more generally). | Thank you for this question. The prospect theory stems from the idea that losses loom larger than gains, hence it is also known as loss aversion theory. People tend to overweight small probabilities to guard against losses. Example would be insurance. When choosing among several alternatives, people avoid losses and optimize for sure wins because the pain of losing is greater than the satisfaction of an equivalent gain.  We have also elaborated the discussion based on your inputs. Thank you for your valuable feedback. |
| 8. | Conclusion  1. You say that vaccinations are the “only” tool to curb pandemic spread, and this is not true. Other protective health measures (e.g., hand washing, mask wearing, etc.) are also effective tools to curb pandemic spread. It might be the case that vaccination is one of the strongest tools to curb pandemic spread, in which case you should offer a citation for this point. | We have made the suggested changes and added a citation to support the statement.  Thank you very much Reviewer A for your constructive comments and suggestions for improvements. |

**Reviewer B Comments**

In this well written paper the authors aimed to investigate the effect of framing vaccine communication message with gain and loss framing based on the Theory of Planned

Behavior. Following comments may be useful.

| **#** | **Specific Comments** | **Corrections Made** |
| --- | --- | --- |
| 1 | Why authors tried to assess perceived threat. This concept is not brought in the TPB. Please explain it. | Thank you for this observation.  Considering the context of the COVID 19 pandemic, its prolonged nature and lower fatality rate made people become optimistically biased of their chances of contracting the virus. This not only lowered their risk perception towards the virus, but also made them hesitant to take up the vaccination (Chu, 2021).  Furthermore, based on past pandemic behaviours, as the mild nature of majority of the cases and the considerable degree of already existing immunity in older age groups becomes clear, initial worries of a moderately serious pandemic fades, just like in the 1968 pandemic (Cowling et al., 2010).  Therefore, we hypothesized perceived threat in the research model, as it is essential to understand the perception of risk towards the COVID 19 virus among millennials in India, to predict their vaccination uptake. Thus extending the theory of planned behaviour for the current scenario. |
| 2 | I suggest to remove hypothesis. | Taking into consideration both the reviewers’ comments, we have made improvisation to the hypotheses to make them specific to what was measured. |
| 3 | The discussion section need to be extended by well interpretations. | We have now elaborated the discussion based on your inputs. Thank you for your valuable feedback. |
| 4 | I also suggest to address to infodemics in the COVID-19 era. | Thank you for your feedback. We have incorporated the following lines in the introduction and literature review:  “Moreover, with false information and fake news surrounding COVID 19 being on the rise especially on social media (Allahverdipour, 2020) COVID 19 vaccine hesitancy could increase amongst the millennials.”  “The importance of coherent and sound information cannot be denied to gain public trust surrounding COVID 19 vaccinations especially in this era of infodemic (Allahverdipour, 2020).” |
| 5 | The following paper may be useful in explain infodemic:  Global Challenge of Health Communication: Infodemia in the Coronavirus Disease (COVID-19) Pandemic.J Educ Community Health. 2020;7(2): 65-67. doi: 10.29252/jech.7.2.65 | Thank you for your valuable comments and suggestion to refer to this paper. This is very relevant to this paper and we have cited it accordingly. |
